# The Participation of Acetyl Phosphate, a Microbial and Host Metabolite, in the Regulation of the Calcium Balance in Mitochondria and Cells

**DOI:** 10.3390/ijms27021007

**Published:** 2026-01-20

**Authors:** Natalia V. Beloborodova, Alexey V. Berezhnov, Nadezhda I. Fedotcheva

**Affiliations:** 1 Federal Research and Clinical Center of Intensive Care Medicine and Rehabilitology, 25-2 Petrovka St., Moscow 107031, Russia; nvbeloborodova@yandex.ru; 2 Institute of Cell Biophysics, Russian Academy of Sciences, 3 Institutskaya St., Pushchino 142290, Russia; alexberezhnov@pbcras.ru; 3 Institute of Theoretical and Experimental Biophysics, Russian Academy of Sciences, 3 Institutskaya St., Pushchino 142290, Russia

**Keywords:** acetyl phosphate, mitochondria, calcium, mitochondrial permeability transition pore, neuroblastoma cells, astrocytes, hyperphosphatemia, sepsis, purinergic signaling

## Abstract

Acetyl phosphate (AcP) is a microbial metabolite acting as a link between cell metabolism and signaling, providing the survival of bacteria in the host. AcP was also identified as an intermediate of pyruvate oxidation in mammalian mitochondria and was found in the human blood in some severe pathologies. The possible contribution of circulating AcP to the maintenance of the physiological or pathological states of the body has not been studied. Since AcP can function as a donor of phosphate groups, we have examined *in vitro* the influence of AcP on calcium signaling in mitochondria and cells by measuring the membrane potential and the calcium retention capacity of mitochondria by selective electrodes and by assaying the cell calcium signaling by Fura-2AM fluorescent radiometry. AcP was shown to induce a concentration-dependent increase in the mitochondrial resistance to calcium ion loading both in the control and in the presence of ADP. This effect was especially pronounced when mitochondria were incubated in a phosphate-free medium; under these conditions, AcP strongly raised the membrane potential and increased the rate of calcium uptake and the calcium retention capacity several times. Moreover, AcP induced similar changes in human cells when calcium signaling was activated by ATP, to a greater extent in neuroblastoma cells than in astrocytes. In the presence of AcP, a tendency for an increase in the amplitude and a decrease in the continuance of the ATP-induced calcium response was observed. These changes are probably associated with the activation of calcium buffering by mitochondria due to the delivery of phosphate during the hydrolysis of AcP. The results show that AcP is involved in the regulation of the Ca^2+^ balance in cells by activating the accumulation of calcium ions by mitochondria, especially under phosphate deficiency. A shift in calcium signaling mediated by AcP supplementation may be caused by hyperphosphatemia, which is now considered as one of basic contributors to cellular dysfunction and progression of various diseases, including sepsis.

## 1. Introduction

Acetyl phosphate (AcP) is a microbial metabolite involved in the acetate kinase/phosphate acetyltransferase pathway of the central bacterial metabolism. Depending on bacterial growth conditions, the reversible synthesis of AcP or ATP is accomplished by this pathway [1,2]. In parallel, AcP can function as a donor of both acetyl and phosphoryl groups [1,2,3,4]. As a donor of acetyl groups, AcP mediates the nonenzymatic acetylation of the enzymes of the central bacterial metabolism, including the glycolysis and the tricarboxylic acid cycle [4,5]. Moreover, among other effects, AcP provides the survival of bacteria in the host via the modulation of chemotaxis, virulence, and antibiotic resistance [6,7]. As a donor of phosphoryl groups, AcP participates in signal transduction, phosphorylating the response regulators in bacterial two-component signal transduction, which allows bacteria to perceive diverse stimuli and respond to them [8,9]. Therefore, in bacteria, AcP was suggested to act as a link between metabolism and cell signaling [10]. It was shown that the response regulator can be phosphorylated not only by the membrane-bound histidine kinase, but also by low-molecular-weight phosphodonors, such as AcP [10,11]. It was found that the two-component regulatory system of *Streptococcus pneumoniae* can be activated by AcP-dependent phosphorylation and can affect a large variety of physiological processes, including ß-lactam resistance, competence development, bacteriocin production, as well as host colonization and virulence [12].

As was shown, in bloodstream infections, bacteria activate the production of AcP due to the involvement of pyruvate oxidase, which promotes the bacterial aerobic growth and traffic across host barriers [13]. Pyruvate oxidase has been implicated in pneumococcal pathogenesis in animal models of pneumonia and sepsis, especially at a high level of blood glucose as a source of pyruvate [13]. It is important that AcP was found in the human blood in some severe pathologies. A high level of AcP in the blood was recorded in spinal cord pathologies and interpreted as a sign of mitochondrial breakdown [14]. In mammalian mitochondria, AcP was identified as an intermediate in the tricarboxylic acid cycle, which forms during the oxidation of pyruvate and rather rapidly degrades, probably by acyl phosphatase [15]. AcP was supposed to be involved in an uncharacterized metabolic pathway in human mitochondria, possibly via phosphorylation or acetylation processes [15]. Recently, AcP was found among human blood metabolites associated with metabolic disturbances in cancer [16]. As follows from these data, the source of AcP both in bacteria and the host is the oxidation of pyruvate. Thus, the origin of AcP present in the blood can be either microbial, associated with bacterial overgrowth or impaired microbiota metabolism, or mitochondrial, associated with impaired metabolism or the degradation of mitochondria. Consequently, the presence of AcP in the blood reflects most likely pathological changes. As a donor of acetyl groups, AcP may mediate the nonenzymatic acetylation of the enzymes. Indeed, the hyperacetylation of mitochondrial proteins and enzymes was revealed in a number of pathologies, including sepsis and inflammation [17,18]. We have previously found that AcP moderately suppresses the oxidative phosphorylation, succinate dehydrogenase activity, and the opening of the mitochondrial permeability transition pore (mPTP), thus increasing the resistance to the calcium load [19].

Calcium is crucial for normal physiological function. Typically, disorders of blood calcium levels are associated with a poor prognosis in various diseases. It is well-established that calcium homeostasis is disrupted in sepsis, though the direction of this imbalance can vary. Clinical evidence suggests that both hypercalcemia and hypocalcemia are associated with an increased risk of mortality in septic patients [20]. Furthermore, calcium-sensing receptors play a significant role in modulating sepsis-induced organ dysfunction. These receptors exhibit tissue-specific protective effects in certain tissues while promoting inflammatory responses in others. Therefore, elucidating these dual effects and their underlying signaling pathways is crucial and could facilitate the development of targeted therapies for sepsis-related organ damage [21]. In addition to the participation in acetylation, AcP may serve as a source of phosphate groups, undergoing the hydrolysis by acyl phosphatase, which cleaves the carboxyl–phosphate bond [22]. It is known that phosphate plays a vital role in diverse biological processes, including calcium signaling, membrane integrity, energy production, and others. Cells respond to changes in the phosphate concentration in their environment by adjusting the phosphate uptake and transforming the biochemical processes [23]. Both excess and deficit of phosphate may cause various health issues such as pathological calcification, oxidative stress, cell death, and abnormal signal transduction [24]. The function of AcP as a phosphate donor has not been considered before. In the present study, we examined the influence of AcP on Ca^2+^ signaling in mitochondria and cells, considering AcP as a phosphorylating agent and a donor of phosphate groups. The effect of AcP on the Ca^2+^-induced mPTP opening was tested in the control and in the presence of ADP, a physiological metabolite and simultaneously an inhibitor of the mitochondrial pore. Since mitochondria sense a rise in the cytosolic calcium and perform the calcium buffering, we investigated the influence of AcP on the calcium signaling in cells. For this purpose, the calcium signaling in cells was activated by ATP supplementation, and the magnitude of the ATP-induced calcium response (the amplitude and the time) in the presence of AcP was assessed.

## 2. Results

**Figure 1:**
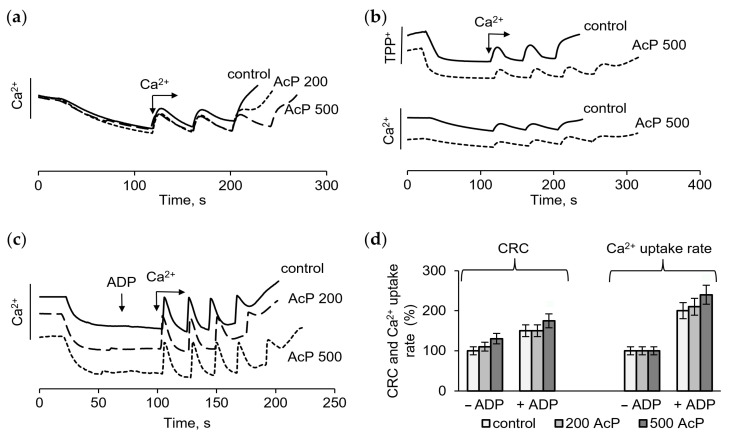
Effect of AcP on the calcium retention capacity and the membrane potential in the control and in the presence of ADP in a phosphate-enriched medium. The influence of AcP at different concentrations on the calcium uptake (**a**), the membrane potential (**b**) in the control and in the presence 25 µM ADP (**c**) during successive additions of CaCl_2_ at 50 µM each; changes in the calcium retention capacity (CRC) and the calcium uptake rate depending on the concentration of AcP (**d**); the incubation medium contained 125 mM KCl, 1.5 mM KH_2_PO_4_, and 15 mM HEPES (pH 7.25).

The influence of AcP as a phosphate donor on the mitochondrial calcium uptake was assessed by measuring the membrane potential and the calcium retention capacity (CRC) in standard and phosphate-free incubation media. The role of AcP in the protective effect of ADP on the resistance of mitochondria to a calcium overload was also determined. As shown on Figure 1, in a standard medium with phosphate, calcium ion loading in the course of successive additions led to the opening of the nonspecific mitochondrial pore (mPTP), recorded by the release of accumulated calcium ions (Figure 1a), and a drop of the membrane potential, as estimated by the release of accumulated TPP^+^ (Figure 1b). AcP (at a concentration of 200 μM and higher) moderately increased the ability of mitochondria to accumulate calcium ions before the pore opening. At concentrations of 200–500 μM, AcP increased the CRC by one next 50 μM calcium addition compared to the control (Figure 1a,b). Both the drop in the membrane potential and the release of calcium ions show that, in the presence of 500 μM AcP, the threshold calcium concentrations required to open the pore increased by 20–25% (Figure 1b). In the presence of ADP, an inhibitor of mPTP, the CRC and the calcium uptake rate increased approximately twofold in the control and further increased slightly with the addition of AcP (Figure 1c). Thus, in a phosphate-enriched incubation medium, AcP moderately increases these parameters both in the control and in the presence of ADP (Figure 1d).

**Figure 2:**
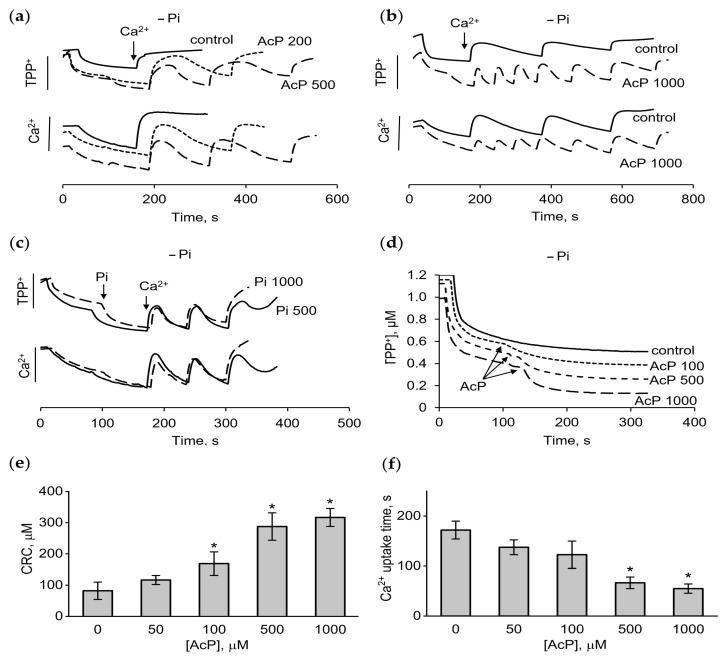
Effect of AcP on the membrane potential, calcium uptake, and calcium retention capacity in phosphate-free medium. Changes in the membrane potential and calcium uptake in the presence of AcP (**a**,**b**) or phosphate (**c**) during successive additions of CaCl_2_ at 50 µM each; the influence of AcP at indicated concentrations on the membrane potential (**d**); the influence of AcP at different concentrations on calcium retention capacity (CRC) (**e**) and the calcium uptake time (**f**); the incubation medium contained 125 mM KCl and 15 mM HEPES (pH 7.25); an asterisk (*) indicates the values ± S.D. that differ significantly from the control values (*p* < 0.05).

However, in a phosphate-free medium, AcP caused strong changes in these parameters. As shown, in this incubation medium, mitochondria either did not accumulate calcium at all (Figure 2a) or accumulated only 50–100 μM calcium supplement (Figure 2b). The addition of AcP in the concentration range of 200–1000 μM increased the accumulation of calcium ions by two to four times (Figure 2a,b). Simultaneously, a sharp increase in the rate of calcium uptake was observed. It is important that the addition of AcP led to a rise in the membrane potential, which is evident from the decrease in the concentration of TPP^+^ in the medium in response to the addition of AcP (Figure 2a). Adding phosphate to the medium produced approximately the same response. As shown in Figure 2c, phosphate also induced an increase in the membrane potential and the CRC. However, elevating the phosphate concentration from 500 to 1000 μM did not stimulate a further increase in the CRC, which distinguishes its effect from that of AcP. Additionally, differences were observed in the magnitude and the rate of increase in the membrane potential in response to the addition of AcP and phosphate. While phosphate at concentrations of 500 and 1000 μM increased the membrane potential equally (Figure 2c), AcP further improved this parameter with increasing concentration (Figure 2d). At the same concentration of added phosphate and AcP (500 μM each), the rate of the increase in the membrane potential differed more than twofold in favor of inorganic phosphate. Thus, AcP and inorganic phosphate have a unidirectional influence on the membrane potential and calcium uptake, differing in the intensity of the effect. Figure 2e,f show an increase in the CRC and the rate of calcium uptake depending on the AcP concentration in a phosphate-free medium.

**Figure 3:**
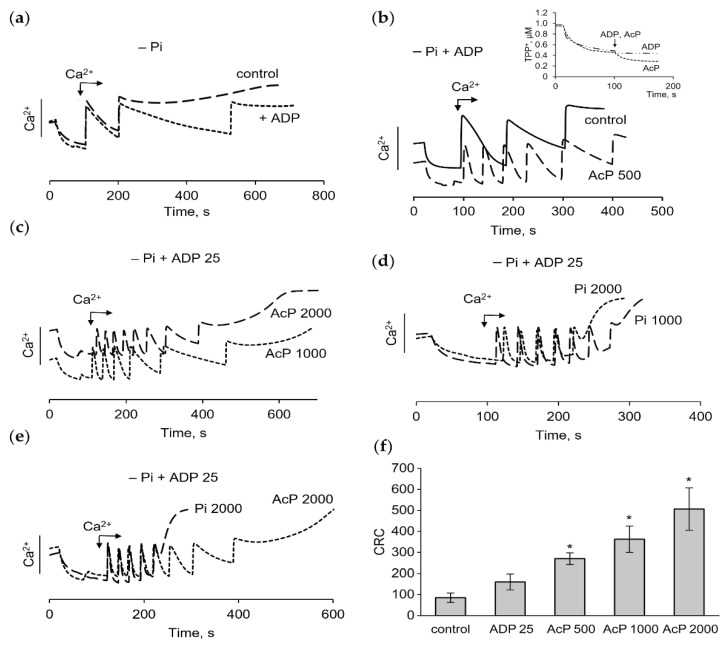
Effect of AcP on the calcium uptake, calcium retention capacity and the membrane potential in phosphate-free medium in the presence of ADP. The influence of ADP (25 µM) on the accumulation of calcium ions during successive additions of CaCl_2_ at 50 µM each in the control (**a**) and in the presence of AcP (**b**,**c**) or in the presence of phosphate (**d**) at indicated concentrations; differences in the effects of phosphate (2000 µM) and AcP (2000 µM) on the pore opening during calcium loading (**e**); changes in CRC (µM) at different concentrations of AcP in phosphate-free medium with ADP (**f**); an asterisk (*) indicates the values ± S.D. that differ significantly from the control values (*p* < 0.05).

The influence of AcP on the pore opening in a phosphate-free medium with the supplementation of ADP, an inhibitor of mPTP, was also tested. In this medium, ADP (25 μM) increased the CRC by one 50 μM calcium addition, so that the next portion of calcium did not accumulate and the pore did not open (Figure 3a). In the absence of phosphate, ADP did not affect the membrane potential, unlike AcP (Figure 3b, insert). However, ADP greatly enhanced the effect of AcP on the CRC and the rate of calcium uptake. As shown in Figure 3b,c, under these conditions, AcP in the concentration range from 500 to 2000 μM increased the CRC and the calcium uptake rate by several times. Under the same conditions, phosphate in physiological concentrations (1–2 mM) also increased the CRC and the calcium uptake rate, but with some differences from the action of AcP. Phosphate mediated a rapid opening of the pore, when the threshold calcium concentrations were reached, and decreased the CRC as its concentration increased to 2 mM (Figure 3d). These differences are clearly visible in Figure 3e, which shows the opening of mPTP during successive additions of calcium in the presence of 2 mM AcP or 2 mM phosphate. Figure 3f shows an increase in the CRC depending on the AcP concentration in a phosphate-free medium in the presence of ADP. As is evident, the effect of AcP under these conditions is more pronounced than in a phosphate-enriched medium (Figure 1) and in the absence of ADP (Figure 2).

**Figure 4:**
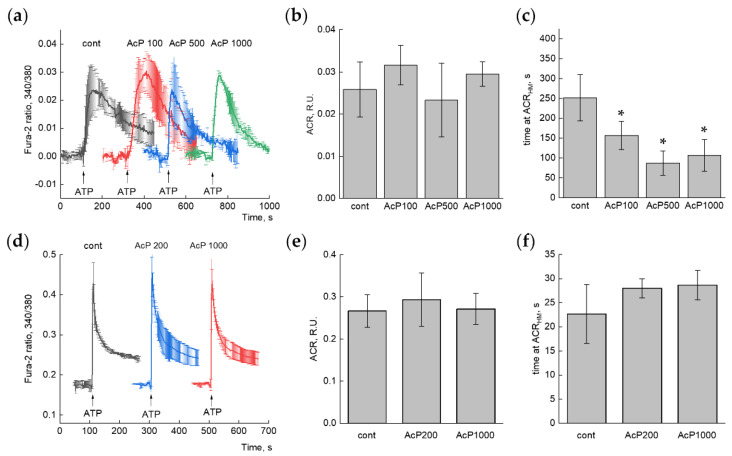
Effect of AcP on the ATP-induced calcium response in SH-SY5Y neuroblastoma cells (**a**–**c**) and primary rat astrocytes (**d**–**f**) cells. (**a**,**d**)—Averaged Ca^2+^-signals in control conditions and in the presence of different concentrations (in μM) of AcP. Each curve is the average (+/− SD) of 3–4 independent experiments. *n* = 965 (cont), 542 (AcP 100), 419 (AcP 500), 460 (AcP 1000) for (**a**) and >300 cells in each experiment for (**d**). (**b**,**e**)—Quantitative analysis of the amplitudes of calcium responses (ACRs), (**c**,**f**)—Quantitative analysis of the duration of calcium peak (time at the half-maximal amplitude). An asterisk (*) indicates the values ± S.D. that differ significantly from the control values (*p* < 0.05).

The presence of AcP in the blood suggests its primary action on cells. Since mitochondria sense a rise in the cytosolic calcium and perform the calcium buffering, we investigated the influence of AcP on calcium signaling in cells. For this purpose, the calcium signaling was activated by ATP supplementation, and the magnitude of the ATP-induced calcium response in the presence of AcP was assessed. The amplitude and the time of calcium signaling were evaluated. Since the ATP-dependent calcium signal is well induced in different cell types, the human-derived cell line SH-SY5Y and primary rat hippocampal astrocytes were tested in our experiments. ATP supplementation stimulated a transient increase in the cell calcium concentration, which varied for individual cells. We analyzed an average calcium response (Figure 4a,d) from all cells in the experiment (usually more than 140 cells). AcP itself did not change the basal calcium concentration and the amplitude of cytosolic calcium response both in SH-SY5Y and astrocytes (Figure 4b,e). However, in SH-SY5Y cells, the duration of the averaged ATP-induced calcium signal tended to decrease in the presence of AcP (Figure 4c). This effect enhanced with the increasing concentration of AcP from 100 to 1000 μM. In astrocytes, on the contrary, AcP caused some increase in signal duration (Figure 4f).

## 3. Discussion

Our results show a new effect of AcP associated with the regulation of phosphate homeostasis and, as a consequence, with the regulation of the calcium signal in mitochondria and cells. According to our data, AcP moderately increases mitochondrial resistance to calcium loading in a phosphate-enriched medium and strongly activates the calcium uptake and the calcium retention capacity in a phosphate-free medium. These effects of AcP are fully reproduced in neuroblastoma cells, manifesting themselves in the activation of ATP-induced Ca^2+^ signaling. At the same time, the effect of AcP as a phosphate donor is mediated by the preceding rupture of the carboxyl-phosphate ester bond, which implies the participation of the enzymatic or spontaneous hydrolysis of AcP. This is probably related to the differences observed in the action of AcP and inorganic phosphate in experiments on mitochondria. These differences can be explained by modes of phosphate delivery: AcP is hydrolyzed constantly, yielding a gradual rise of phosphate, while the addition of phosphate creates an immediate high matrix-phosphate load, which can sensitize the mPTP.

Inorganic phosphate is the main intracellular anion capable of entering the mitochondria and an essential component for Ca^2+^ buffering by mitochondria. It is well known that excessive accumulation of free calcium is a key factor in inducing the mPTP opening since the repetitive mitochondrial Ca^2+^ loading causes a gradual increase in the concentration of mitochondrial calcium, leading to a drop in the membrane potential and the release of Ca^2+^. Inorganic phosphate is a major participant in maintaining the trans-matrix pH gradient and the buffering of matrix Ca^2+^ through the formation of calcium phosphate granules [25,26]. It is believed that the calcium-phosphate buffer system maintains free Ca^2+^ at a steady-state level, providing a greater Ca^2+^ loading, not impeding the Ca^2+^ uptake, and not affecting the efflux system [27]. Our data on the influence of AcP as a phosphate donor on the resistance to calcium loading are consistent with this proposition. However, the testing of AcP in these modes revealed two other important factors that are essential for Ca^2+^ buffering and the regulation of pore opening. These is a rise in the membrane potential and in the rate of calcium uptake in response to the addition of AcP, both effects being dependent on its concentration. These effects were generally reproduced when replacing AcP with phosphate. Although the increase in the membrane potential induced by a high concentration of phosphate has been noted previously [28,29], this fact has not attracted sufficient attention. It was shown that increasing the phosphate concentration from zero to 1000 μM raised the membrane potential from the initial 160 to 190 mV [30]. Moreover, in these studies, an increase in the production and release of reactive oxygen species (ROS) was observed in parallel with a rise in the membrane potential; additionally, it was noted that an increase in the concentration of phosphate alone was sufficient to significantly augment ROS generation in mitochondria. Interestingly, these data are now considered to explain the toxic effects of high phosphate concentrations in pathological conditions, in which the intracellular phosphate level increases for various reasons, such as due to the hydrolysis of the high-energy phosphate bond [30,31,32,33,34,35].

In this context, mitochondria are the main target of the toxic effect of phosphate at high concentrations. This conclusion is supported by the experimental data on cells, which showed that the elevated extracellular phosphate by itself increased the mitochondrial membrane potential, superoxide generation, membrane damage, and cell death, and moreover, that these alterations were removed either by antioxidants, or protonophores, or the inhibitors of mitochondrial phosphate transporters [28,32,34]. The accumulation of calcium ions leads to opposite changes related to a drop in the membrane potential and the opening of the mitochondrial pore. As follows from our data, even at high concentrations, AcP exhibits protective properties, increasing the resistance to the calcium load and the rate of calcium uptake. This effect of AcP differs from the known effect of high concentrations of inorganic phosphate, at which the calcium retention capacity of mitochondria generally decreases [26,36]. These differences may be associated with the hydrolysis of the high-energy phosphate bond of AcP, which can occur with the participation of acyl phosphatase [20,37,38] or, spontaneously, under alkaline conditions and with the availability of metal ions [39]. Acyl phosphatase hydrolyzes the carboxyl–phosphate bonds of different acyl phosphates, including the intermediates of glycolysis, membrane pumps, the tricarboxylic acid cycle, and urea biosynthesis [37,40]. Recently, it has been demonstrated that acyl phosphatase plays a role in glioma progression via the regulation of the calcium and phosphate balance in glioma cells [38].

A new aspect of the participation of AcP in maintaining the physiological or pathological states of the body is related to its influence on the purinergic activation of calcium signaling in cells. As follows from our data, in mitochondria, AcP promotes the phosphate-dependent accumulation of calcium ions and greatly increases the rate of calcium uptake. Very similar changes were observed in our experiments on neuroblastoma cells upon the activation of calcium signaling by ATP. In the presence of AcP, the signal duration significantly decreased, by more than 40%. The faster calcium response, compared to the control, is due apparently to the activation of calcium accumulation by mitochondria. This assumption is consistent with the concept that the high level of phosphate directly improves the mitochondrial Ca^2+^-buffering capacity, especially under pathophysiological conditions, when the calcium clearance by plasma membrane transporters, which pump Ca^2+^ outside the cell, as well as the reticulum Ca^2+^-ATPase, which pumps Ca^2+^ into the reticulum, is insufficient, as it occurs during ischemia–reperfusion and other injuries [41]. While in isolated mitochondria, this effect of AcP suggests a protective role via the prevention of the pore opening, the functional significance of the activation of ATP-induced signaling by AcP in cells is a controversial subject. As is known, extracellular ATP predominantly functions as a signaling molecule through the activation of purinergic receptors, which play a critical role in immune cell migration, chemotaxis, and cytokine release [42,43,44]. Purinergic receptors exist in cells of nearly all types in the body and are involved in a number of cellular processes such as inflammation, immune activation, neuronal transmission, proliferation, and apoptosis. ATP is released from tissues in necrosis, hypoxia, and apoptosis as well as from immune cells during infection and acts as a “danger” signal inducing a response of the host to infections, inflammation, and organ injury [44]. It was also found that the stimulation of monocytes by bacterial lipopolysaccharides led to the accumulation of extracellular ATP [45]. Additionally, the increased level of ATP in the serum was recorded in septic patients and was considered as a possible biomarker of sepsis [42,46]. The activation of purinergic receptors after ATP binding promotes the influx of Na^+^ and Ca^2+^ and the efflux of K^+^, following which the increase in the intracellular Ca^2+^ concentration activates various downstream cellular processes [47]. It is believed that a specific signaling response (phosphorylation, or gene transcription, or migration) depends on the duration, location, strength, and frequency of the Ca^2+^ signal [48,49]. It was revealed in model experiments on microglia that small Ca^2+^ transients accompany migration, while large and sustained transients give rise to cytokine production [50]. Based on these data, it can be assumed that the activation of ATP-induced signaling by AcP is probably associated with a stimulation of specific inflammatory reactions due to phosphate delivery or phosphorylation processes (as in the signal transduction in bacteria), which requires a more detailed study. The contribution of AcP to the ATP-induced signaling suggests a new role for microbial metabolites in the regulation of inflammation and sepsis. It is important to emphasize that purinergic signaling, especially the signaling mediated by the nonselective adenosine triphosphate-gated cation channel P2X7, is involved in the disturbance of the blood–brain barrier permeability [51] and the disruption of the intestinal epithelial barrier [52]. It has been found that selective agonists/antagonists of purinergic receptors modulate the permeability of the blood–brain barrier and reduce the brain cell apoptosis and sepsis-associated encephalopathy caused by lipopolysaccharides [51,53]. Also, systemic P2X7 blockade downregulated sepsis-induced inflammatory responses and attenuated intestinal barrier dysfunction in mice treated with the antagonist [52]. It is believed that P2X7 receptors can be converted into a non-selective channel or a pore, which is permeable to small molecules in the conditions of the continued delivery of ATP [51,52,54]. These receptors are thought to be involved in the barrier disruption and, if so, AcP may be involved in this process by activating the ATP-induced signaling. As has been shown, the human neuronal SH-SY5Y cell line expresses the P2X7 receptor [55,56]. At the same time, although several purinergic receptors are present in almost all cells, their activation depends on the receptor type and the ATP concentration, which is specific to each type. In addition to these factors (presence of certain receptors, dependence on ATP concentration), the degree of the influence of AcP may also vary depending on the appropriate hydrolysis conditions. These three factors may determine the differences in the effects of AcP on the ATP-induced signaling in neuroblastoma cells and astrocytes in our experiments. Despite these differences and peculiarities, our data indicate that AcP contributes to the regulation of ATP-induced calcium signaling, predominantly by influencing the duration of signals.

Our data are consistent with the assumptions that phosphate in itself can represent a signal molecule regulating multiple factors necessary for diverse biological processes and that a phosphate-sensing mechanism may operate in various organs, detecting changes in the serum or the local phosphate concentration [23,57]. In this connection, it is interesting to note that the physiological concentrations of phosphate vary over a small range. Inorganic phosphate is maintained in equilibrium in the range of 0.8–1.4 mM Pi, while hyperphosphatemia can be classified according to the level of serum phosphate in the patient as mild (1.44–1.76 mM), moderate (1.76–2.08 mM), or severe (>2.08 mM) [34,58]. Hyperphosphatemia is one of the main causes of morbidity and mortality in patients with chronic kidney disease and sepsis [59,60]. As is known, excessive intake of dietary phosphate may result in various health issues, such as dental, cardiovascular, and kidney diseases, as well as diabetes and cancer, which are associated with high phosphate-induced pathological calcification, oxidative stress, cell death, and abnormal signal transduction [31]. Our data suggest that AcP can increase the phosphate level in blood, cells, and mitochondria, which may be beneficial in hypophosphatemia or toxic in hyperphosphatemia.

When considering the bacterial or mitochondrial origin of AcP, it is important to emphasize that this metabolite is found in the blood in severe pathologies [14,16]. Its presence in the blood reflects probably functional changes in mitochondria or metabolic preferences in bacteria. In both bacteria and mitochondria, the precursor of AcP is pyruvate. Recently, pyruvic acid and AcP were identified among microbiota metabolites, predominantly enriched during dyslipidemia, and were associated with all gut microbial taxa [61]. In this study, AcP was elevated in hypercholesterolemia and positively associated with a high level of the low-density lipoprotein cholesterol in persons tested [61]. As an end product, AcP is formed in bacteria with the participation of three different enzymes, the activation of which depends on anaerobic or aerobic growth conditions. During anaerobic growth, acetate kinase, which catalyzes the formation of AcP from acetate and ATP, and phosphate acetyltransferase, which catalyzes the conversion of acetyl-CoA into AcP, are involved in the formation of AcP. Under aerobic conditions, pyruvate oxidase catalyzes the oxidative decarboxylation of pyruvate to AcP with the formation of hydrogen peroxide as a by-product. Both products are now considered important in bacterial survival and their trafficking across the blood–brain barrier [13,62]. While these three enzymes are strictly bacterial, acyl phosphatase, which hydrolyzes AcP, is widely distributed in diverse organisms, from bacteria to higher eukaryotes, including the humans [63]. In mammalian cells, acyl phosphatase (ACYP) is localized in the cytosol and widely occurs in different tissues. It can catalyze the hydrolysis of not only low-molecular-weight substrates, such as acetyl phosphate, but also the phosphorylated intermediates of various ATPases. Acyl phosphatase 1 (ACYP1) was shown to be associated with tumor initiation and progression coupled with the expression of metabolism-related genes [24]. ACYP1 markedly enhanced the proliferation, invasion, and migration of hepatocellular carcinoma cells, increasing the expression of genes related to aerobic glycolysis, in particular, lactate dehydrogenase [24]. As was found, in glioma cells, ACYP2 functions as an oncogene via the regulation of intracellular Ca^2+^ homeostasis [38]. It was noted that ACYP2 is distributed in both the cytoplasm and the nucleus in hepatocellular carcinoma cells, and its overexpression leads to the inhibition of the proliferation and metastasis of these cells and an enhancement of the apoptosis [37]. At the same time, there are only limited data regarding the functional role of the bacterial enzyme. As has been shown, acyl phosphatase from *Staphylococcus aureus* has high hydrolase activity, which is strongly dependent on the substrate concentration and is inhibited by high concentrations of ATP, which acts as a competitive inhibitor of the enzyme [40]. In *Escherichia coli*, the overexpression of acyl phosphatase was accompanied by an increase in the intracellular steady-state calcium concentration [64]. It can be assumed that the interaction or/and the switch-over of pathways of AcP synthesis and hydrolysis have a functional significance in the transition from anaerobic to aerobic conditions, affecting the survival of bacteria in the host.

Limitations: In the context of sepsis-related disorders, it is important to emphasize that the mechanisms underlying the disruption of the integration of endogenous and microbial metabolisms in favor of pathogenic bacteria are still unknown [65,66]. Sepsis is a life-threatening pathological condition that is broadly related to bacteria but does not have a single specific causative agent [66,67]. The dual origin of AcP (microbial vs. mitochondrial) reflects the importance of such an interaction. It should be noted that the mechanism of AcP formation in mitochondria is still unknown. Presumably, AcP is predominantly of bacterial origin, as its concentration in bacteria can reach 3 mM [3]. Our data indicate potential targets of AcP action, namely mPTP and purinergic receptors. It is important that both systems share common regulatory mechanisms, involving ATP and phosphate. The results obtained highlight the need for a more detailed study of the mechanisms of AcP action in experimental animal models of sepsis, as well as in endothelial and intestinal cells associated with barrier functions.

## 4. Materials and Methods

Lithium potassium acetyl phosphate, Fura-2AM, adenosine diphosphate, CaCl_2_, and others reagents were from the Sigma–Aldrich Corporation (St. Louis, MO, USA).

### 4.1. Animals and Isolation of Rat Liver Mitochondria

The study was conducted in accordance with the ethical principles formulated in the Helsinki Declaration on the care and use of laboratory animals. Manipulations were carried out by the certified staff of the Animal Department of the Institute of Theoretical and Experimental Biophysics Russian Academy of Sciences and approved by the Commission on Biomedical Ethics of ITEB RAS (N30/2025, 3 March 2025). Experiments were performed on male rats of the Wistar line. Male (6- to 8-week-old) rats were kept under the same conditions in air-conditioned and ventilated rooms at 20–22 °C with a 12 h/12 h light–dark cycle.

Mitochondria were isolated from the liver using a standard method of differential centrifugation in a medium containing 300 mM sucrose, 1 mM EGTA, and 10 mM Tris-HCl buffer (pH 7.4). A sample of mitochondria was washed with the isolation medium without EGTA, suspended in a medium of the same composition, and stored on ice.

### 4.2. Measurement of the Membrane Potential and the Calcium Retention Capacity of Mitochondria

The voltage difference across the membrane was determined by the distribution of the lipophilic cation tetraphenylphosphonium (TPP^+^), whose concentration in the medium was recorded with a TPP^+^-selective electrode (Nico, Moscow, Russia). A decrease in the concentration of positively charged TPP in the incubation medium indicates a high membrane potential, while an increase in its concentration in the medium reflects a drop in a membrane potential. The accumulation of Ca^2+^ in the mitochondria was recorded with a Ca^2+^ selective electrode as the change in the calcium concentration in the external medium in response to successive CaCl_2_ additions with a “Record 4” device (Nico, Moscow, Russia). The calcium retention capacity (CRC) of mitochondria was determined by the ability of mitochondria to accumulate successive additions of calcium ions to the threshold concentration necessary for the opening of the nonspecific mitochondrial pore. The standard incubation medium contained 125 mM KCl, 1.5 mM KH_2_PO_4_, and 15 mM HEPES (pH 7.25); mitochondrial supplement was 1 mg protein per mL. Other experimental conditions are indicated in the captions to the figures. The figures show the data of typical experiments performed in at least five replicates with different samples of mitochondria.

### 4.3. Assay of Cell Calcium Signaling

Object. Human neuroblastoma SH-SY5Y cells were grown to ~70% confluence on 25 mm round cover glasses, placed in 35 mm Petri dishes, in a CO_2_ incubator in DMEM containing 10% FBS. Primary rat astrocytes were isolated from the hippocampus of P2-3 male pups, as previously described [68], with modifications. Briefly, the hippocampi were removed, cut with scissors, and left in a papain-containing (0.8%) Versene solution for 24 min. Then, the enzyme was washed out by centrifugation, and the bathing solution was replaced by DMEM with 10% FBS. Cells were grown in a CO_2_ incubator for 10 days in a 25 cm^2^ flask. Then, cells were seeded on 25 mm round cover glasses and left in the CO_2_ incubator for 2 days before the experiment.

### 4.4. Staining

Cells were washed from culture medium with Hank’s balanced salt solution (HBSS), pH 7.4, and loaded with a Fura-2AM fluorescent radiometric calcium-sensitive probe for 40 min at 37 °C. Then, cells were washed twice in dye-free HBSS and mounted in a chamber.

### 4.5. Experimental Procedure

Experiments were carried out at 28 °C. An experimental chamber with a cell culture was fixed on a table of an Axiovert 200M microscope (Carl Zeiss AG, Oberkochen, Germany). Dual-channel (excitation at 340 and 380 nm) images were obtained using a set of 21HE light filters (Carl Zeiss AG, Oberkochen, Germany) and a 20× objective with an interval of 1 frame per 2–3 s. The volume of the incubation solution was 1 mL. During the addition of AcP and ATP, uniform and rapid stirring of the compounds was provided. One minute after the start of recording, either a control change-over of the solution was carried out (“cont” experiments), or 100–1000 µM AcP was added (“AcP” experiments). After another 5 min, ATP (20 µM) was added, and the recording was continued for 5 min. Each type of the experiment was performed in triplicate.

### 4.6. Image Analysis

The time-lapse images obtained were analyzed using the Image J/FiJi (NIH, Bethesda, MD, USA) software. Origin 2016 (OriginLab Corporation, Northampton, MA, USA) and GraphPad Prism 8 (GraphPad Software Inc., Boston, MA, USA) were used to plot the graphs. Each time lapse contains two channels, C340 and C380, i.e., time series images of the cell culture when Fura-2 was excited at 340 and 380 nm. After initial background subtraction, basic 3 × 3 smoothing was applied to the time lapse image. The C340 channel was then divided by C380, and signal measurements were performed on the resulting 32-bit image. ROIs were created corresponding to individual cells. For astrocytes, the number of cells in the experiment exceeded 300, and more than 140 cells were analyzed in each experiment with neuroblastoma cells. No additional calibration of Fura-2 signal was performed. In the case of SH-SY5Y the resting level was subtracted from the entire trace to bring it to the zero starting value. 

### 4.7. Statistical Analysis

The data presented represent either the mean ± standard deviation (S.D.) of three to five experiments or typical traces from identical experiments. After passing the Shapiro–Wilk normality test, statistical differences between the experimental and control groups were determined using a one-way analysis of variance (ANOVA) with Dunnett’s post hoc test. Differences were considered significant at *p* < 0.05. Statistical analysis was performed in GraphPad Prism 7.05, Origin 2018 and MS Excel were used for graphical visualization.

## 5. Conclusions

Thus, the influence of AcP as a source of phosphate can extend to vital cellular functions, such as the opening of the mitochondrial pore and the buffering of calcium excess, the activation of purinergic signaling and the selection of the inflammatory response, as well as changes in the phosphate level in the serum, cytosol, and mitochondria. Variations in these processes are closely interrelated and during inflammation and sepsis are associated with the mitochondrial dysfunction. A number of studies have shown that bloodstream infections and mitochondrial dysfunctions are accompanied by the secretion of bioactive metabolites released by them into the systemic circulation [9,65,66,69]. It has been found that in the norm (in healthy people with a “healthy” microbiota), the products of microbial metabolism enter the systemic bloodstream and are the end metabolites, while microbial metabolites associated with sepsis can be intermediates [70]. Under aerobic conditions, the bacterial production of AcP increases, which may enhance hyperphosphatemia and modulate the inflammatory response via purinergic calcium signaling.

## Data Availability

The original contributions presented in this study are included in the article. Further inquiries can be directed to the corresponding author.

## Abbreviations

The following abbreviations are used in this manuscript:

AcP	acetyl phosphate
ACR	amplitudes of calcium response
ADP	adenosine diphosphate
CRC	calcium retention capacity
mPTP	mitochondrial permeability transition pore
Pi	inorganic phosphate
TPP^+^	tetraphenylphosphonium ion
